# Isolated Cardiac Hydatid Cyst Causing Complete Heart Block

**DOI:** 10.7759/cureus.11945

**Published:** 2020-12-07

**Authors:** Ismahane Lahmidi, Mohamed Boutaybi, Jamal El Ouazzani, Noha Elouafi, Zakaria Bazid

**Affiliations:** 1 Cardiology, Mohammed I University/Mohammed VI University Hospital, Oujda, MAR

**Keywords:** cardiac hydatidosis, complete heart block, pace maker

## Abstract

Cardiac hydatidosis is an unusual disease and the interventricular septum is infrequently involved. It can cause various complications because of rupture and embolization. Interventricular septal cysts, may in rare conditions, induce symptoms corresponding to compression of the conduction pathway such as atrioventricular block. In this report, we present an uncommon case of cardiac echinococcus located in the basal part of the interventricular septum presenting as complete heart block managed by medical treatment and implantation of a permanent pacemaker.

## Introduction

Hydatid disease, also known as hydatidosis, is engendered by infection with Echinococcus granulosus; it results in the constitution of one or more hydatid cysts situated frequently in the liver and lungs, and rarely in the kidneys, bones, spleen, muscles, and central nervous system. Cardiac involvement is very rare and it is encountered in only 0.5% to 2% of patients with hydatid disease [1,2]. Different segments of the heart can be affected; the location of a hydatid cyst in the interventricular septum is just 4% of cardiac cases [1,2]. Cyst growth is slow, only 10% of cardiac hydatid cyst patients are symptomatic [1]. Because of the lack of specific symptoms, the diagnosis of cardiac hydatid cyst can be challenging. We report a case of isolated cardiac hydatid cyst causing complete atrioventricular block which is infrequently observed in clinical presentation.

## Case presentation

**Figure 1:**
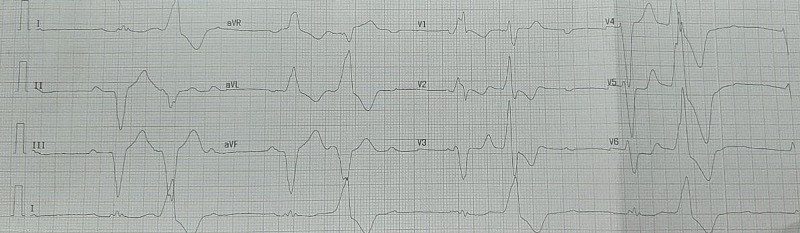
Electrocardiogram showing a complete atrioventricular block

**Figure 2:**
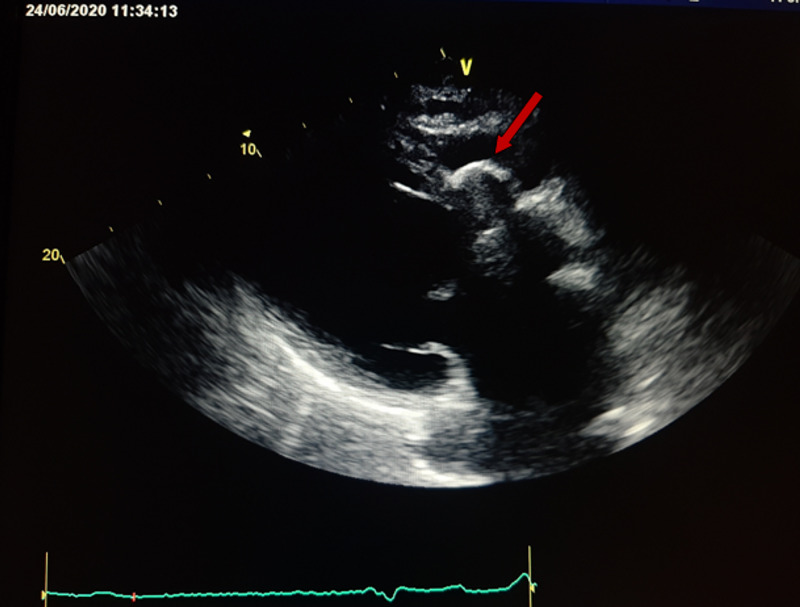
Transthoracic echocardiography showing cyst in the basal part of interventricular septum in the parasternal long-axis view

**Figure 3:**
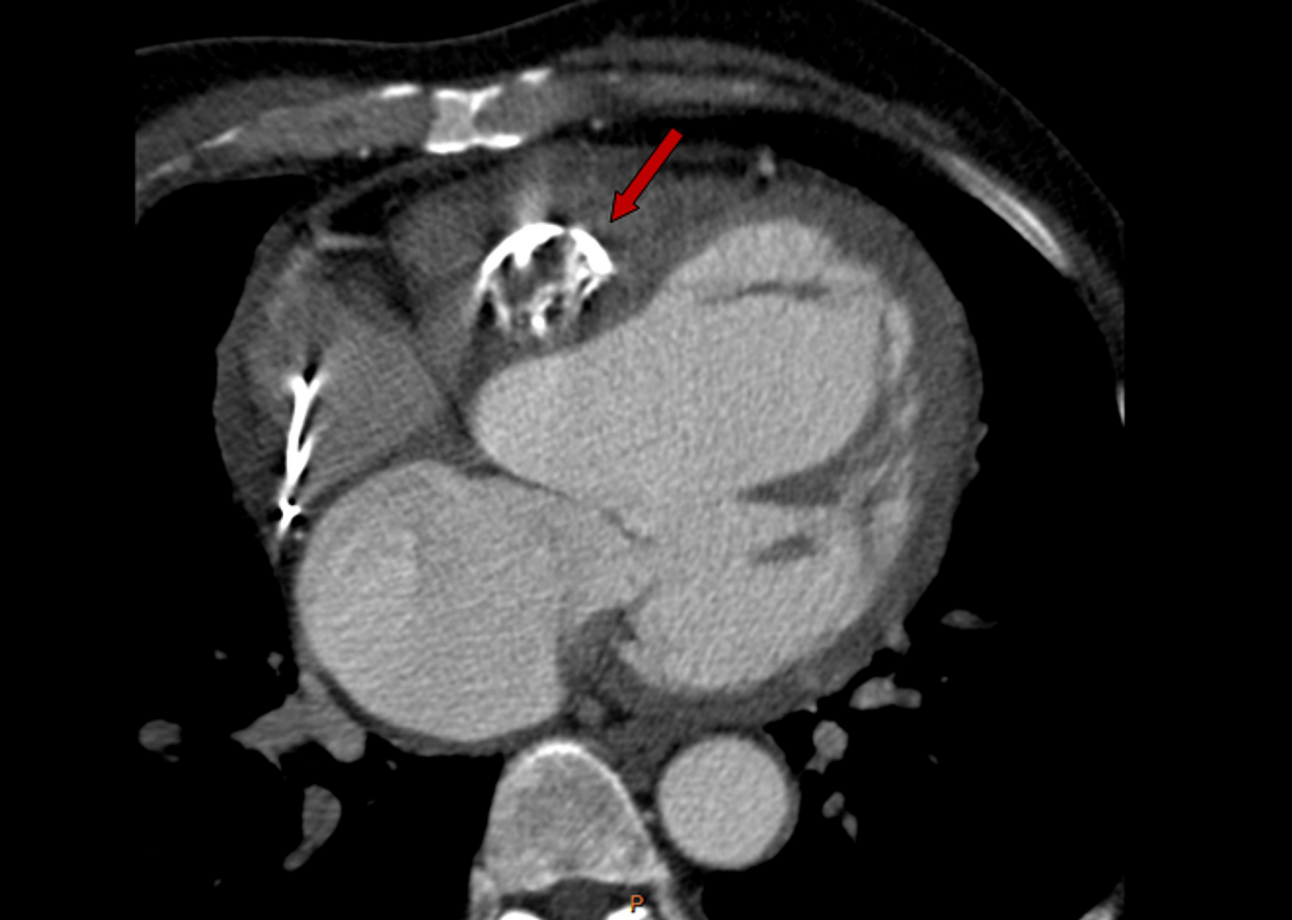
Computed tomography imaging showing a cyst located in basal interventricular septum with multiple calcifications

A 67-year-old male patient was admitted to our hospital with chief complaints of asthenia and dyspnea. His past medical history was unremarkable. In his physical examination, blood pressure was recorded at 110/70 mmHg and his heart rate was 40 bpm. A complete atrioventricular block was revealed by an electrocardiogram (ECG) (Figure 1). Routine laboratory test results were normal. Transthoracic echocardiography showed a cystic formation of size 3.7 × 2.5 cm situated in the basal part of the interventricular septum (Figure 2). We noted also a reduced left ventricular ejection fraction (40%). Cardiac computed tomographic examination showed a well-defined cystic lesion in the interventricular septum, suggestive of hydatid cyst (Figure 3). Serologic tests for echinococcal infection were strongly positive. Thus, cardiac hydatid cyst with intraseptal involvement diagnosis was made.

Owing to the size and location of the calcified cyst, surgical resection was not considered in order to minimize the risk of rupture of the contents; medical treatment with albendazole was initiated. A permanent pacemaker was implanted. The patient was discharged with albendazole treatment and was doing well at four months follow up.

## Discussion

Human hydatidosis is an infection generated by the larvae of Echinococcus granulosus that remains endemic in Morocco. Primary hosts are dogs and other carnivores; some of the herbivorous and omnivorous animals are intermediate hosts, while humans are accidental hosts. Frequently affected sites by hydatid cysts are the liver and lungs, however, any body organ may be at risk for concern. Cardiac hydatid cysts are an uncommon condition, with a rate ranging from 0.5% to 2%. The most involved part of the heart is the left ventricular owing to its maximum blood supply (60%), followed by the right ventricle (10%), pericardium (7%), left atrium (6%-8%), right atrium (3%-4%), and interventricular septum (4%) [1,2]. Williams reported the first case of cardiac localization in 1936 [3]. The clinical presentation ranges from asymptomatic to sudden death. Cardinal symptoms are chest pain, dyspnea, palpitations, and cough, they differ in accordance with the number, size, and site of the cysts [4]. As was described in our case, a cyst located in the basal septum close to the conduction system can induce atrioventricular block by compression of the conduction pathway.

Echocardiography is a performant and sensitive modality of choice to diagnose cardiac hydatid cyst. It allows a detailed characterization of the cysts, their size, number, localization, and their effect, and furthermore, any eventual complications. Computed tomography and MRI are essential for establishing the diagnosis, they furnish a detailed assessment of the lesions and their relationship with other structures [5]. In our case, the diagnosis was made by transthoracic echocardiography and confirmed by computed tomography assessment.

Standardized therapeutic management cannot be practicable owing to the diversified character of cardiac hydatidosis, however, surgery is still the treatment of choice for cardiac hydatid cysts, as well as in asymptomatic cases, to prevent life-threatening complications [6]. In some circumstances, medical treatment may be the only alternative, it could be indicated for small or calcified cysts, in elderly patients, or when surgery is contraindicated or refused by the patient [7]. In our case, because of the size, the calcified character of the cyst and increased operative risks, antihelminthic treatment was preferred and a permanent pacemaker was implanted.

## Conclusions

To sum up, clinical presentations of cardiac hydatid disease are nonspecific and might mimic diverse cardiac diseases according to the location of the cyst. Cardiac hydatidosis should be considered in patients who present from areas where the disease is endemic.
